# Therapeutic vaccination against fibronectin ED-A attenuates progression of metastatic breast cancer

**DOI:** 10.18632/oncotarget.2628

**Published:** 2014-10-24

**Authors:** Julia Femel, Elisabeth J.M. Huijbers, Falk Saupe, Jessica Cedervall, Lei Zhang, Pernilla Roswall, Erik Larsson, Helena Olofsson, Kristian Pietras, Anna Dimberg, Lars Hellman, Anna-Karin Olsson

**Affiliations:** ^1^ Department of Medical Biochemistry and Microbiology, Science for Life Laboratory, Uppsala University, Biomedical Center, Uppsala; ^2^ Department of Immunology, Genetics and Pathology, Uppsala University, Rudbeck Laboratory, Uppsala; ^3^ Department of Medical Biochemistry and Biophysics, Karolinska Institutet, Stockholm; ^4^ Department of Laboratory Medicine, Lund University, Medicon Village AB, Lund; ^5^ Department of Cell and Molecular Biology, Uppsala University, Sweden

**Keywords:** ED-A, immunization, tumor vasculature, therapeutic, cancer vaccine

## Abstract

Therapeutic vaccination targeting self-molecules is an attractive alternative to monoclonal antibody-based therapies for cancer and various inflammatory diseases. However, development of cancer vaccines targeting self-molecules has proven difficult. One complicating factor is that tumor cells have developed strategies to escape recognition by the immune system. Antigens specifically expressed by the tumor vasculature can therefore provide alternative targets. The alternatively spliced extra domain-A and B (ED-A and ED-B) of fibronectin are expressed during vasculogenesis in the embryo, but essentially undetectable under normal conditions in the adult. However, these domains are re-expressed during tumor angiogenesis and matrix remodeling, which renders them highly interesting for targeted cancer therapies.

Using the MMTV-PyMT transgenic model of metastatic mammary carcinoma, we show that tumor burden can be significantly decreased by immunization against ED-A in a therapeutic setting. Furthermore, we found that in mice carrying anti-ED-A antibodies the number of metastases was reduced. ED-A immunization increased infiltration of macrophages and compromised tumor blood vessel function. These findings implicate an attack of the tumor vasculature by the immune system, through a polyclonal antibody response. We conclude that tumor vascular antigens are promising candidates for development of therapeutic vaccines targeting growth of primary tumors as well as disseminated disease.

## INTRODUCTION

Therapeutic vaccination targeting self-molecules could provide a cost-efficient alternative to monoclonal antibody-based therapies for cancer and other diseases. Development of cancer vaccines has not yet been successful enough to qualify as a standard therapy in the clinic. The reason for this is probably multifaceted. One obstacle of targeting tumor-associated antigens is the strategies of immune evasion employed by tumor cells. An alternative approach is therefore to direct the immunotherapy against the tumor vasculature, since stromal cells in a tumor are more genetically stable than the malignant cells. Moreover, the vasculature is easily accessible for immune cells or antibodies from the circulation and it has been estimated that one endothelial cell supports approximately 100 tumor cells [1, 2]. A second major reason for the absence of therapeutic vaccines in the clinic targeting self-molecules is the lack of sufficiently potent, but non-toxic and biodegradable adjuvants for use in humans. We have in two recent publications [3, 4] demonstrated that it is possible to achieve an efficient immune response against self-antigens using the adjuvant Montanide ISA 720/CpG (MN720/CpG), which is acceptable for clinical use [4]. This finding should significantly aid the development of therapeutic vaccines targeting self-molecules.

**Figure 1:**
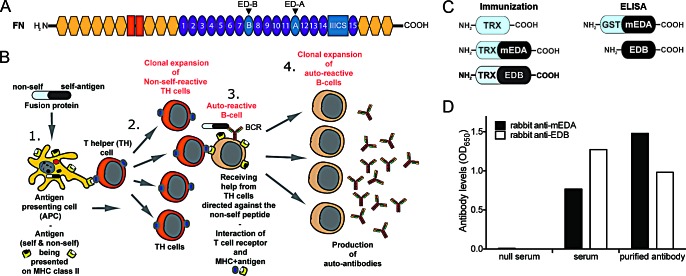
Generation of domain-specific vaccine proteins and anti-sera against ED-A and ED-B (*A*) Schematic illustration of a fibronectin (FN) monomer. The extra domain-A (ED-A) and B (ED-B) of FN are indicated by arrows. (*B*) Schematic presentation of the immunization strategy. 1. Antigen presenting cells (APC) internalize the fusion protein and present peptides from the non-self and self-part on MHC class II. 2. T-helper cells (TH) recognize peptides from the non-self part with their T-cell receptor and are activated. Self-peptides are not recognized due to tolerance mechanisms eliminating self-reactive T-cells during development. 3. Autoreactive B-cells are present in the circulation and via their B-cell receptor (BCR; immunoglobulin) they recognize the self-part of the fusion protein. This results in internalization and degradation of the Ig-bound fusion protein and presentation of peptides from both the self- and the non-self part on MHC class II on the B-cell surface. TH cells previously activated by non-self peptides from the fusion protein can now provide activation help to these auto-reactive B cells presenting the same non-self peptides. 4. The activated auto-reactive B-cells undergo clonal expansion and produce anti-self antibodies. (*C*) Recombinant fusion proteins used for immunization (TRX-containing) and detection of antigen-specific antibodies in ELISA (lacking TRX). (*D*) Detection of rabbit anti-mouse EDA (black bars) and rabbit anti-EDB (white bars) antibodies by ELISA in serum from immunized rabbits, before (serum) or after affinity purification (purified antibody). Null serum obtained before immunization was used as negative control.

We have recently shown that it is possible to efficiently break self-tolerance against the tumor vascular marker ED-B (extra domain-B) of fibronectin. Mice immunized against ED-B in a prophylactic setting displayed reduced functionality of the tumor vasculature and a 70% reduction in tumor growth [5]. ED-B, as well as the extra-domain A (ED-A), are inserted into fibronectin (FN) by alternative splicing (Fig 1A). These extra-domains of FN are expressed during vasculogenesis in the embryo but essentially undetectable under normal conditions in the adult. Both ED-A and ED-B are highly expressed around angiogenic vasculature in various tumor types and show a strong conservation between species [6, 7]. While there is a two amino acids difference between mouse and human ED-A, the ED-B sequence is 100% identical in several species such as mouse, rat, rabbit, dog, monkey and human, which eliminates the need for species-specific vaccines against ED-B. The restricted expression pattern makes ED-A and ED-B highly interesting for targeted cancer therapies, such as therapeutic vaccines.

In our previous study targeting ED-B, we employed a prophylactic immunization strategy inducing immunity before tumor cell inoculation was initiated. The clinical situation will however be different. Patients already diagnosed with cancer, and perhaps also metastatic disease, are in need of efficient therapy. An important question to answer is therefore whether we can achieve suppression of tumor growth in a therapeutic setting. To address this issue we have used the MMTV-PyMT mouse model of metastatic mammary adenocarcinoma, which is considered to closely resemble the step-wise progression, morphology and molecular signature of human breast cancer [8]. Immunization against ED-A was induced after the onset of tumorigenesis and resulted in a significant decrease in tumor burden. This was accompanied by an increased influx of macrophages into the tumor and a decreased functionality of the tumor vasculature with respect to leakiness and perfusion. Importantly, the number of pulmonary metastases was reduced in MMTV-PyMT mice immunized against ED-A. Since metastatic disease remains the primary cause of death for cancer patients, this is a highly relevant finding for the development of drugs targeting disseminated malignant disease.

## RESULTS

### Generation of domain-specific vaccine proteins and anti-sera against ED-A and ED-B

A recombinant protein consisting of the self-antigen to be targeted fused to a foreign (= non-self) part, derived for example from bacteria, is used to break self-tolerance against the target antigen [9]. Combined with an immunostimulatory adjuvant, the fusion protein can induce high anti-self antibody titers. A schematic illustration of the vaccination mechanism is shown in Figure 1B. Bacterial thioredoxin (TRX) was used as the foreign part of the vaccine proteins. TRX-mouse (m) EDA was cloned and expressed in *E. coli* as previously described for TRX-EDB [5] (Fig 1C). Recombinant TRX, without fusion partner, was generated for immunization of control groups. To detect antibody responses specific for mouse ED-A and ED-B in ELISA, recombinant proteins lacking the TRX-part were produced in a similar way. Another fusion partner, glutathione-S-transferase (GST) was added to EDA, since this domain could not be stably produced on its own (Fig 1C).

To generate antibodies against the mouse ED-A and ED-B domains for immunostainings of mouse tissue, we immunized rabbits with the TRX-mEDA (hereafter called TRX-EDA) and TRX-EDB fusion proteins. The rabbit sera showed strong anti-mED-A or anti-ED-B immunoreactivity in ELISA, both before and after affinity purification against recombinant GST-mEDA or EDB, respectively (Fig 1D).

### ED-A is expressed in MMTV-PyMT tumors in a similar pattern as in human breast cancer

**Figure 2:**
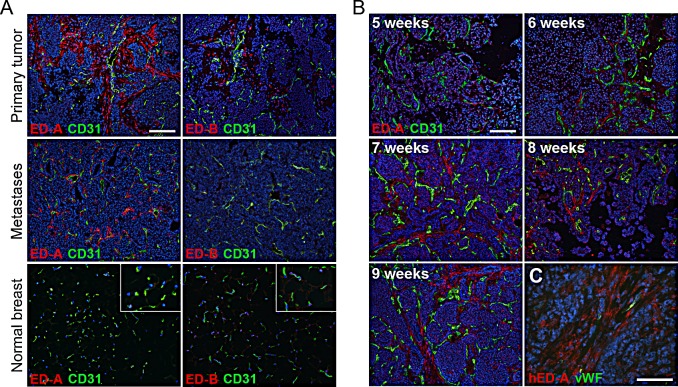
ED-A is expressed both in primary tumors and metastases in the MMTV-PyMT model of mammary carcinoma (*A*) The affinity-purified rabbit anti-mouse ED-A and anti-ED-B antibodies were used for immunostainings (red) of primary MMTV-PyMT mammary carcinomas (upper panels) and lung metastases (middle panels) from female 13-week-old MMTV-PyMT positive mice. Normal breast tissue (lower panels) was derived from 13-week-old MMTV-PyMT negative female mice. Insets show an enlarged area of the immunostained healthy tissue. (*B*) Immunostainings of breast tissue from MMTV-PyMT positive female mice of different ages (5 to 9 weeks) for ED-A (red). Tissue was co-stained for CD31 to visualize blood vessels (green). (*C*) Immunostaining for ED-A in human ductal mammary carcinoma tissue using an affinity purified anti-ED-A antibody directed against the human sequence (hED-A, red). Co-staining for von-Willebrand factor (vWF) was performed to visualize blood vessels (green). Nuclei were counterstained with Hoechst. Scale bars: 100 μm.

An important aspect in cancer vaccine development is to find vaccines that are efficient when an individual has already been diagnosed with cancer, since this reflects the situation in the clinic. This goal has proven significantly more difficult compared to prophylactic strategies. To address the therapeutic potential of a vascular targeting vaccine in a relevant tumor model, we analyzed expression of ED-A and ED-B in the transgenic MMTV-PyMT model of metastatic mammary carcinoma, using the affinity-purified antibodies described above. In this model the polyoma middle-T antigen (PyMT) is expressed under the control of the mouse mammary tumor virus promoter (MMTV) [10]. The MMTV-PyMT mice gradually develop adenocarcinomas in all mammary epithelia, which are clearly palpable by 8-10 weeks of age. Tumor formation and progression is characterized by four stages: hyperplasia, adenoma/mammary intra-epithelial neoplasia, and early and late carcinoma [8]. Other similarities to the human situation are the gradual loss of steroid hormone receptors and ß1-integrin, which is associated with overexpression of ErbB2 and cyclin D1 [11]. Moreover, the MMTV-PyMT model is characterized by a high incidence of pulmonary metastases detectable from around week 12-13. ED-A is reported to be present at high levels in human breast carcinomas and metastases [12], while ED-B is less abundant in this tumor type. Immunostainings of MMTV-PyMT breast tumors and pulmonary metastases from 13 week old mice showed that ED-A was prominently expressed around the tumor vasculature, both in the primary tumor and the metastases (Fig 2A). However, non-vascularized metastases had no detectable ED-A staining. Expression of ED-B was also seen in the vasculature of primary MMTV-PyMT tumors, but in contrast to ED-A no expression was found in the lung metastases. Neither ED-A nor ED-B was expressed in the healthy mammary tissue (Fig 2A). Immunostaining of human ductal breast carcinoma tissue, using an anti-human ED-A antibody produced “in-house” in the same way as the mouse-specific antibodies, confirmed a prominent stromal expression of ED-A (Fig 2C). To analyze the kinetics of ED-A expression in the MMTV-PyMT model, breast tissue from positive female mice of different ages (5 to 9 weeks) was immunostained for ED-A. Expression was visible in early hyperplastic lesions of 5-week old mice and increased with age and tumor progression (Fig 2B). Altogether, these findings establish ED-A as a relevant target for therapeutic immunization in the MMTV-PyMT model for metastatic breast cancer.

### TRX-EDA vaccination induces anti-ED-A antibodies and reduces tumor growth in a therapeutic setting

**Figure 3:**
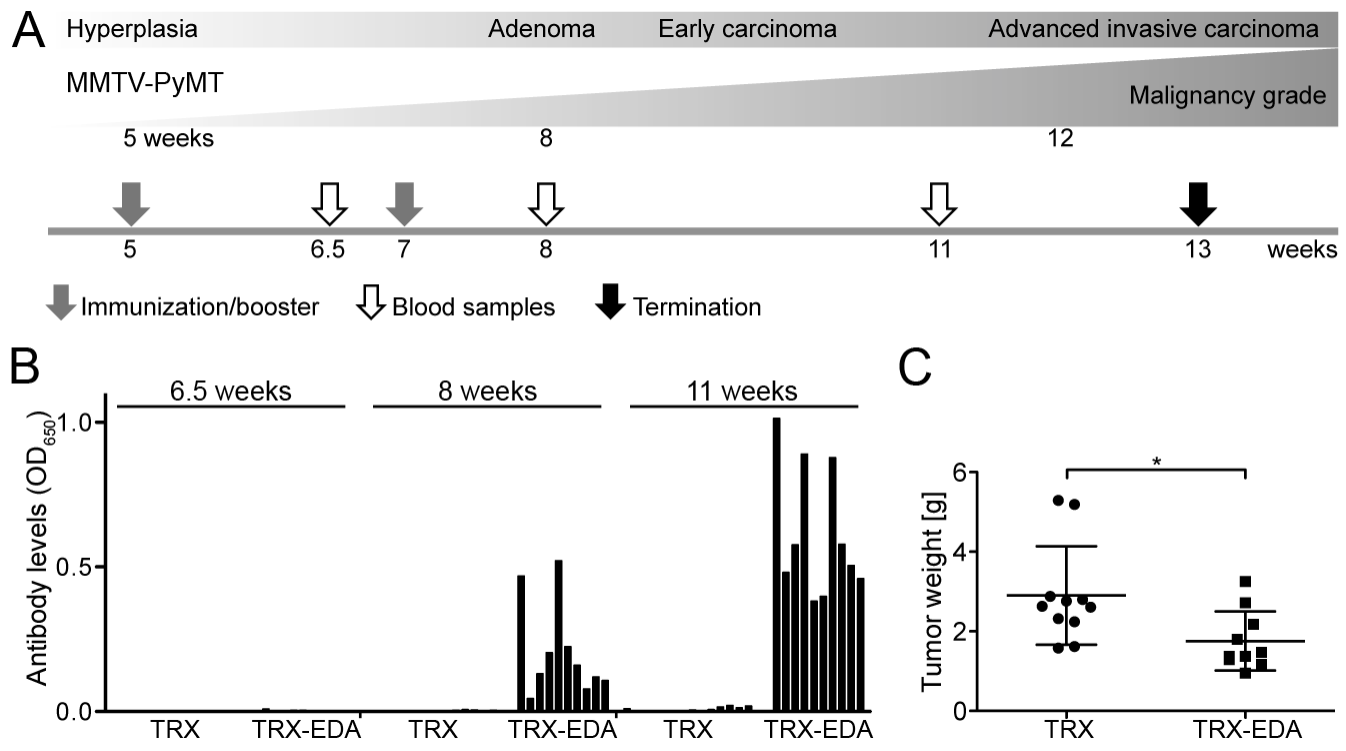
Vaccination with TRX-EDA induces anti-ED-A antibodies and reduces tumor growth in a therapeutic setting (*A*) Illustration of tumor progression in the MMTV-PyMT model of metastatic mammary carcinoma. Arrows indicate time-points for vaccinations, blood sampling and termination. (*B*) Analysis of the presence of anti-ED-A antibodies by ELISA in serum collected from TRX-vaccinated (control) and TRX-EDA vaccinated mice at different time points (week 6.5, 8 and 11). Bars represent individual animals. (*C*) Total tumor weight in MMTV-PyMT mice immunized with TRX (n = 11) or TRX-EDA (n = 10), p = 0.0124. Data represent mean ± standard deviation (SD).

A major challenge in addressing the effect of therapeutic cancer vaccines in murine tumor models is the rapid tumor growth in mice compared to humans, combined with the fact that induction of a robust immune response takes several weeks. After the first vaccination a second injection (booster) is required after a few weeks in order to induce detectable antibody titers against the antigen. In the MMTV-PyMT model, hyperplasia in mammary tissue is detectable from week five and tumors are palpable from eight weeks of age. To elicit an anti-ED-A antibody response at the time tumors have progressed to carcinomas, five week-old MMTV-PyMT mice received a first vaccination with TRX-EDA (n = 10) or the control protein TRX (n = 11) in the presence of the adjuvant MN720/CpG. A booster vaccination was given at week seven. Blood samples were collected at week 6.5, 8 and 11 for analysis of anti-ED-A antibodies in ELISA (Fig 3A). At week 6.5 no anti-ED-A antibodies were detectable in any of the two groups of mice (TRX or TRX-EDA; Fig 3B), which is consistent with the lag-phase of a humoral response. However, after the booster, mice vaccinated with TRX-EDA showed detectable levels of anti-ED-A antibodies at week 8, which increased significantly until week 11 when 100% of the mice had responded with antibody production against ED-A (Fig 3B). No anti-ED-A reactivity was detected in serum samples from mice immunized with the control protein TRX. At week 13 the mice were euthanized and the total tumor burden was determined. As can be seen in Fig 3C, the tumor weight at this time was significantly reduced in mice immunized against ED-A (Fig 3C), showing the potential of the applied strategy. In fact, the difference between the two groups with respect to tumor burden is underestimated in the presented graph, since three mice from the control group had to be euthanized before week 13 due to high tumor burden and were thus not included in the final analysis. In contrast, all TRX-EDA vaccinated mice remained throughout the study. In summary, these data show that it is possible to suppress tumor growth with a therapeutic vaccine targeting tumor vessels - also in situations where aggressive tumorigenesis has already been established.

### ED-A immunity attracts immune cells and impairs functionality of tumor blood vessels

**Figure 4:**
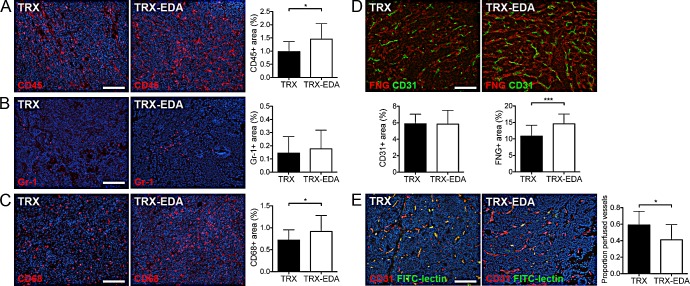
Vaccination against ED-A attracts immune cells and decreases the functionality of tumor blood vessels (*A*) Representative immunostainings for the leukocyte marker CD45 (red). Bar graph depicts quantification of CD45^+^ area (TRX n = 20, TRX-EDA n = 19, p = 0.0129). (*B*) Representative immunostainings for the neutrophil marker Gr-1 (red). Bar graph depicts quantification of Gr-1^+^ area (TRX n = 21, TRX-EDA n = 20, p = 0.1792). (*C*) Representative immunostainings for the macrophage marker CD68 (red). Bar graph depicts quantification of CD68^+^ area (TRX n = 18, TRX-EDA n = 20, p = 0.0452). (*D*) Representative immunostainings for fibrinogen (FNG, red). Tissue was co-stained with CD31 to visualize blood vessels (green). Left bar graph depicts quantification of CD31^+^ area (TRX and TRX-EDA n = 19, p = 0.4835) and the right graph depicts quantification of FNG^+^ area (TRX and TRX-EDA n = 19, p = 0.0006). (*E*) Representative images of FITC-lectin perfused blood vessels (green). Total amount of blood vessels was visualized by immunostaining for CD31 (red). The proportion of FITC-lectin perfused vessels per 20X field is depicted in the bar graph (TRX n = 14, TRX-EDA n = 16, p = 0.0134). Nuclei were counterstained with Hoechst. Data represent mean values ± SD, n represents the number of tumors analyzed. Scale bars: 100 μm.

To address to what extent the presence of the tumor vascular targeting anti-ED-A antibodies was able to attract immune cells to the tumor tissue, we analyzed the amount of leukocytes in breast carcinomas from TRX and TRX-EDA vaccinated mice, by immunostaining for the pan-leukocyte marker CD45. As can be seen in Fig 4A, the amount of CD45-positive leukocytes was significantly higher in mice carrying anti-ED-A antibodies. To further investigate which celltype/s were responsible for this increase we analyzed the amount of Gr-1 (mainly neutrophils) and CD68 (macrophages) expressing cells. The number of tumor infiltrating neutrophils in this model was relatively low and no difference between TRX and TRX-EDA vaccinated mice was detected. In contrast, the number of CD68-positive macrophages infiltrating the tumor was higher and also significantly increased in TRX-EDA vaccinated individuals (Fig 4C).

We next investigated how the polyclonal anti-ED-A antibody response affected the ED-A-expressing tumor vasculature. The total amount of CD31-positive tumor blood vessels was similar in the two groups of mice (Fig 4D). However, the functionality of the tumor vasculature in mice with anti-ED-A antibodies was compromised as judged by the increased amount of extravasated fibrinogen, indicative of vascular leakage (Fig 4D). Furthermore, the proportion of FITC-lectin perfused blood vessels was significantly reduced in TRX-EDA vaccinated mice (Fig 4E). In summary, our data suggest that the decrease in tumor volume is caused by reduced vessel function, as a result of an antibody-mediated immune response against the ED-A expressing tumor vasculature.

### Immunization against ED-A attenuates lung metastasis

**Figure 5:**
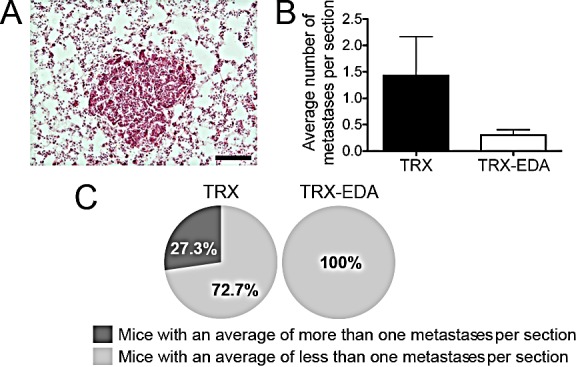
Immunization against ED-A attenuates metastasis (*A*) Example of lung metastases from a 13-week old MMTV-PyMT positive female. Lung tissue was stained with H&E. (*B*) Quantification of the average number of metastases per analyzed sections showed a reduced number of metastases in TRX-EDA vaccinated mice (TRX n = 11, TRX-EDA n = 10). (*C*) Circle diagrams showing the percentage of mice with an average of more than one metastases per section in each group. Data represent mean value ± standard error of the mean (SEM). Scale bar: 100 μm.

To analyze the effect of the ED-A vaccine on metastasis, we sectioned the left lung lobe of 13 week-old mice at every 200 μm. The tissue was stained with hematoxylin and eosin (Fig 5A) and the average number of metastases per section counted. As can be seen in Fig 5B, there was a clear reduction in the number of metastatic foci in the lungs of TRX-EDA immunized mice. Almost a third of the MMTV-PyMT mice in the control-immunized group (TRX) showed more than an average of one metastases per section, while none of the ED-A immunized mice displayed this amount of lung metastases (Fig 5C). This finding demonstrates that anti-ED-A antibodies can efficiently target not only the primary tumor but also the developing metastases.

## DISCUSSION

In this study we show that a therapeutic vaccination strategy against cancer can efficiently suppress growth of solid tumors by directing an immune response towards their vasculature. An important aspect to address is the therapeutic potential of a cancer vaccine, i.e. the efficacy of the vaccine when tumorigenesis is already ongoing, since this represents the clinical situation. A challenge to address therapeutic effects in pre-clinical models is the fact that many subcutaneous models in syngeneic mice reach maximum allowed size within a month or less, which leaves little time to elicit an immune response after detection of the tumor. Furthermore, the ability of the vaccine to suppress formation of metastases after a primary tumor has been detected is of significant value, since the majority of cancer related deaths is caused by disseminated disease. To address these questions we analyzed the effect of a vaccine targeting the tumor vascular antigen ED-A in the transgenic MMTV-PyMT model of mammary carcinoma. This transgenic model of multi-step carcinogenesis shows several important similarities to human ductal carcinoma, forms spontaneous lung metastases and allows for a longer therapeutic window than conventional subcutaneous tumor models. Immunization of MMTV-PyMT mice against ED-A reduced tumor burden in a therapeutic setting and suppressed metastasis. From a clinical perspective these are two highly relevant findings, since a tumor may already have spread to distant sites at the time of diagnosis.

All mice vaccinated with TRX-EDA developed anti-ED-A antibodies after the booster vaccination at week seven. This demonstrates the efficacy of the immunization technique, which is based on the use of a fusion protein consisting of a self- and non-self part, combined with an adjuvant. Lack of sufficiently potent, but at the same time non-toxic and biodegradable adjuvants in the clinic is a limiting factor for development of therapeutic vaccines targeting self-molecules. We have previously characterized the immunostimulatory properties of the squalene-based adjuvant Montanide ISA 720/CpG (MN720/CpG) in comparison to the “golden standard” of preclinical studies, Freund's adjuvant. MN720/CpG was at least as potent or even superior to Freund's with respect to several analyzed parameters such as antibody titers, kinetics of the response and affinity of the induced antibodies [4]. In the present study MN720/CpG was used for all immunizations and hence confirm the ability of the adjuvant to aid the induction of an antibody response against a self-antigen. MN720/CpG has recently been included in a phase I clinical trial and found to be well tolerated [13], which should enable introduction of this immunization strategy to the clinic.

The number of macrophages infiltrating the tumors was increased and the vascular function impaired in animals with anti-ED-A antibodies. These data are in agreement with an immune attack against the tumor vasculature. Antibodies binding to the ED-A domain in the fibronectin molecules present in the extracellular matrix will form immune complexes that attract immune cells, such as macrophages. These will attempt to phagocytose the antibody-antigen complexes. However, since ED-A is not a soluble but tissue-bound antigen, the macrophages are unable to efficiently engulf the immune complexes. This will induce “frustrated phagocytosis”, a process associated with the release of lysosome contents (lysozyme, collagenase, elastase and reactive oxygen species) into the tissue, which results in tissue damage where the antigen is expressed [14].

Immunostainings of tumor tissue from MMTV-PyMT mice showed that ED-A is expressed early during tumorigenesis and in vascularized metastases. It is possible that the anti-ED-A antibodies were already present when the metastases formed, considering that they are established later than the primary tumor. The vasculature of growing metastases may thus have been exposed to an immune attack at an earlier stage of development compared to the primary tumor, explaining the major suppression of metastasis. In humans, where the kinetics of tumor progression is slower than in mice, ED-A vaccination after a cancer diagnosis could therefore potentially be a way to prevent formation of larger vascularized metastases. In addition, it could provide a strategy to prevent disease recurrence after a primary tumor has been removed.

Observations of increased tumor invasiveness and metastasis in pre-clinical models have become a concern when targeting the pro-angiogenic signaling pathways induced by vascular endothelial growth factor (VEGF) with monoclonal antibodies or the tyrosine kinase inhibitor sunitinib [15, 16], currently in clinical use. The data presented here demonstrate that vascular targeting strategies do not necessarily lead to increased invasiveness and metastasis. A distinction between the two strategies is that our approach is not based on removing or inhibiting signals promoting angiogenesis, but instead directs an immune attack towards the tumor vasculature. A speculation could be that the latter situation leaves fewer possibilities for adaptation, which would be an advantage for the patient.

Preclinical studies in murine tumor models using different radiolabelled or cytokine-fused monoclonal antibodies targeting ED-A and ED-B show a high selectivity for targeting of the tumor vasculature and impressive therapeutic responses, which strengthen the feasibility and safety of therapeutic vaccination against the same antigens [17-20]. Currently a number of phase I and II clinical trials are conducted investigating the therapeutic potential of drugs targeting ED-B in cancer patients [21-23]. However, significant costs are associated with monoclonal antibody-based therapies, which limits accessibility for patients. Vaccination with a fusion-protein that is able to induce endogenous antibody production can provide a cost-efficient alternative.

Possible side effects of the vaccine have to be considered and potential risks balanced against benefits for the patient. ED-A and ED-B are highly specific for angiogenic vessels. In the healthy adult body angiogenesis is only associated with very few physiological events, such as wound healing or the female menstrual cycle. Both ED-A and ED-B are transiently expressed during wound healing [24-26]. While it has been reported that mice lacking ED-A displayed defective skin wound healing [27], others have not found this effect [28]. As reported previously, mice immunized against ED-B displayed no impaired wound healing [5]. An important safety aspect of our approach is the reversibility of the immune response induced against the self-antigen [4, 29]. To maintain high antibody titers, two to three immunizations per year are likely to be required.

In conclusion, our data demonstrate that a cancer vaccine targeting ED-A is effective in a therapeutic setting, where it had the capacity to both decrease tumor burden and reduce formation of metastases. Animals carrying anti-ED-A antibodies display an impaired vessel function and increased infiltration of macrophages in the tumors, indicative of an immune response towards the tumor vasculature. The availability of the potent but also biodegradable M720/CpG adjuvant, suitable for the application in humans, and the high degree of conservation of the tumor specific antigens ED-A and ED-B should significantly facilitate introduction of the approach to the clinic.

## MATERIALS AND METHODS

### Ethics Statement

Investigation has been conducted in accordance with the ethical standards and according to the Declaration of Helsinki and according to national and international guidelines and has been approved by the authors' institutional review board. Animal work was approved by the local animal ethics committee (reg. no. C191/10, C198/10, C227/12, C77/13) and performed according to the United Kingdom Coordinating Committee on Cancer Research (UKCCCR) guidelines for the welfare of animals in experimental neoplasia [30], where applicable. Use of human samples from the anonymized biobank material was granted by Uppsala County's ethical committee (Ups 03-412/2003-10-02, Dnr 2010/291/2010-11-17).

### Expression and purification of recombinant proteins

Recombinant proteins were expressed in *Escherichia coli* (*E. coli*) Rosetta gami (DE3) (Novagen; Merck Millipore, Billerica, MA, USA), as previously described for TRX-EDB [5]. cDNAs encoding mouse (m) or human (h) ED-A were cloned in frame with TRX in the pET-21a expression vector, using BamH1 and Xho1. The resulting expression vectors were named pET-21a-TRX-mEDA and pET-21a-TRX-hEDA. Recombinant proteins for detection of antibodies in ELISA were generated by replacing TRX with the sequence encoding for glutathione-S transferase (GST), resulting in pET-21a-GST-mEDA and pET-21-a-GST-hEDA. The addition of a GST-domain was required for stable production of recombinant mouse and human ED-A. For expression of TRX the sequence encoding for EDB was removed from pET-21-a-TRX-EDB, resulting in pET-21-a-TRX. Expression of TRX-containing fusion proteins was induced at 22°C for 16 h, expression of the other proteins was induced at 37°C for 4 h. All recombinant proteins contain a His-tag and were purified using Ni-NTA agarose slurry (Qiagen, Hilden, Germany) and elution with 100 mM to 200 mM imidazole in 20 mM Tris (pH 8.0)/0.1 M NaCl. Imidazole was removed by dialysis against PBS. Protein-containing fractions were dialyzed against PBS using a Spectra/Por CE (cellulose ester) membrane (6 to 8 kDa MW cutoff; Spectrum Laboratories Inc., Rancho Dominguez, CA, USA). Final protein concentration was determined by BCA protein assay (Pierce, Rockford, IL, USA). All proteins were analyzed by mass spectrometry to confirm their identity. The resulting recombinant proteins and their molecular weights were: TRX-EDB (23.1 kDa), TRX-mEDA (24.0 kDa), TRX-hEDA (23.3 kDa), EDB (10.9 kDa), GST-mEDA (37.2 kDa), GST-hEDA (37.2 kDa).

### Generation of rabbit antibodies

Antibodies against mouse ED-A, ED-B and human ED-A were produced in-house. Rabbits were immunized with an emulsion containing 200 μg recombinant TRX-mEDA, TRX-EDB or TRX-hEDA protein in PBS mixed 50:50 with Freund's complete adjuvant (F5881; Sigma-Aldrich, St. Louis, MO, USA), or Freund's incomplete adjuvant (F5506; Sigma-Aldrich) for booster injections. Blood samples were drawn regularly. Antibodies were affinity purified from rabbit serum using Pierce® NHS-activated agarose slurry (26200; Pierce) and 2 ml Pierce® centrifuge columns (89896; Pierce), according to the manufacturer's instructions. GST-mEDA, EDB or GST-hEDA proteins were used as antigens and immobilized to the NHS-activated agarose. Before applying on the Pierce® centrifuge columns, rabbit serum (20 ml) was filtered (0.45 μm filter; Filtropur S 0.45, 83.1826; Sarstedt AB, Helsingborg, Sweden). Bound antibodies were eluted and dialyzed against PBS, using a Spectra/Pore CE membrane. Final antibody concentration was determined by BCA protein assay. The purified antibodies were stored in 10% glycerol at −70 °C.

### Immunization

Five-week old female MMTV-PyMT positive mice (FVB/N background) were immunized with an emulsion containing 100 μg TRX-mEDA or 50 μg TRX (control) and 50 μg CpG oligo 1826 (Sigma-Aldrich) in PBS, mixed 50:50 with Montanide ISA 720 (SEPPIC, Puteaux Cedex, France). Mice received booster injections at seven weeks of age. Blood samples were drawn at age 6.5, 8 and 11 weeks to measure antibody levels. Mice were derived from an in-house breeding colony and successively included into the study at five weeks of age.

### Genotyping of MMTV-PyMT mice

DNA was extracted from tail biopsies and presence of the MMTV-PyMT-transgene was determined using the following primers: forward primer 5′-CGGCGGAGCGAGGAACTGAGGAGAG-3′, backward primer 5′-TCAGAAGACTCGGCAGTCTTAGGCG-3′. The strain was maintained by breeding PyMT-positive males and PyMT-negative females.

### Detection of anti-ED-A antibodies in mouse or rabbit sera

ELISA plates were coated with 8 μg/ml GST-mEDA in PBS (pH 7.4) and blocked with horse serum. Mouse sera were diluted 1:125 in horse serum. Anti-ED-A antibodies were detected with biotinylated goat anti-mouse IgG (H+L) (BA-9200; Vector Laboratories, Burlingame, CA, USA) and streptavidin-horseradish peroxidase (SA-HRP; SA-5004; Vector Laboratories), both diluted 1:500. HRP-activity was detected with TMB substrate (T8665; Sigma-Aldrich) and absorbance was measured at 650 nm. All samples and blanks were assayed as duplicates. To detect anti-mED-A or anti-ED-B antibodies in sera from immunized rabbits, or after affinity purification, an ELISA was performed as described above. Serum and affinity purified antibody was diluted 1:1000 in horse serum.

### Analysis of tumor weight and vessel perfusion

At thirteen weeks of age mice were perfused with 10 ml PBS followed by 10 ml of 2% paraformaldehyde (PFA). Sixteen mice (8 per group) received retro-orbital injection of 150 μl FITC-lectin solution (0.5 mg/ml; Lycopersicon Esculentum, Vector Laboratories) prior to perfusion. Mice were anesthetized with 3% avertin by intraperitoneal injection prior to retro-orbital injection and perfusion. Lung and breast tissue were removed and mammary tumors dissected and weighed. Tumors and lung were cryopreserved in 30% sucrose at 4°C overnight. The FITC signal was enhanced with an anti-FITC antibody (71-1900; Life Technologies, Carlsbad, CA, USA; 1:500).

Six to eight images per tumor were taken at random with a Nikon Eclipse 90i microscope (with a DS-Qi1Mc monochrome CCD camera) (Nikon Instruments Inc., Melville, NY, USA) and NIS Elements AR 3.2 software (Nikon Instruments Inc.) using a 20X objective (Plan Apo 0.75; Nikon Instruments Inc.). The proportion of perfused vessels was counted per 20X field.

### Quantification of metastases

Left lung lobes were cryosectioned completely by taking 10 μm sections in 200 μm-intervals (9-13 sections/lung lobe). The sections were stained with hematoxylin/eosin (H&E; HistoLab Products AB, Göteborg, Sweden). Metastases were counted using a Nikon Eclipse 90i microscope and a 20X objective. Images were taken using a DS-Fi1 color CCD camera (Nikon Instruments Inc.).

### Immunofluorescence and quantification

Cryosections (5 μm) of mammary tumors from immunized mice (TRX-mEDA or TRX) were stained with the following antibodies: anti-fibrinogen (A0080; Dako Sweden AB, Stockholm, Sweden; 1:500), anti-CD31 (553370; BD Pharmingen; BD Biosciences, San Jose, CA, USA; 1:1000), anti-CD45 (553076; BD Pharmingen; 1:200), anti-Gr-1 (Ly-6C and Ly-6G; 553123; BD Pharmingen; 1:1000) and anti-CD68 (MCA1957; AbD Serotec, Oxford, UK; 1:300). Cryosections (7 μm) of mammary tumors and lung tissue from 13-week old MMTV-PyMT positive females and normal breast tissue from MMTV-PyMT negative females mice were stained for ED-A and ED-B using affinity-purified rabbit anti-mED-A antibody (1.2 mg/ml; diluted 1:100) or anti-ED-B antibody (1.5 mg/ml; diluted 1:50). Cryosections were fixed in 100% ice-cold methanol and blocked with 3-5% BSA/PBS, 5% BSA/PBS containing 5% horse serum (CD68 only) or FBS (Gr-1 only). Sections stained for ED-B were treated with 10 000 U/ml N-glycanase before incubation with primary antibody to remove glycosylation. Cryosections of biopsies from human ductal carcinoma were stained for ED-A, using the affinity-purified rabbit anti-hED-A antibody (1 mg/ml; diluted 1:100), and co-stained for von-Willebrand factor (AHP062F; AbD Serotec; 1:100). Fluorophore-conjugated secondary antibodies used were donkey anti-rat Alexa Fluor 488, goat anti-rabbit Alexa Fluor 568 or goat anti-rat Alexa Fluor 568 (all Life Technologies), at 1:1000 dilution. Nuclei were visualized with 1 μg/ml Hoechst 33342 (VWR International, Radnor, PA, USA). Fluoromount-G mounting medium (Southern Biotech, Birmingham, AL, USA) was used to mount stained sections.

Approximately two tumors per mouse were selected and one section per tumor was stained and analyzed for each marker. For quantification, three to 12 pictures of each tumor section, depending on the tumor size, were taken at random with a Nikon Eclipse 90i microscope, using the 20X objective at the same exposure time for each marker, respectively. ImageJ64 10.2 software (National Institutes of Health, Bethesda, MD, USA) was used for quantification of areas (% of 20X field).

### Statistical analysis

Statistical analyses in this study were performed using the non-parametric two-tailed Mann-Whitney U test with GraphPad Prism 5 (5.0c; GraphPad Software Inc., La Jolla, CA, USA). * is defined as p<0.05, ** as p<0.01 and *** as p<0.001.
